# Controllable Optical Bistability and Four-Wave Mixing in a Photonic-Molecule Optomechanics

**DOI:** 10.1186/s11671-019-2893-2

**Published:** 2019-03-01

**Authors:** Hua-Jun Chen, Hong-Wei Wu, Jian-Yong Yang, Xue-Chao Li, Ya-Juan Sun, Yuan Peng

**Affiliations:** School of Mechanics and Photoelectric Physics, Anhui University of Science and Technology, Huainan Anhui, 232001 China

**Keywords:** Photonic-molecule optomechanics, Optical bistability, Four-wave mixing

## Abstract

We theoretically investigate the nonlinear optical phenomena including optical bistability and four-wave mixing (FWM) process in a composite photonic-molecule cavity optomechanical system. The photonic-molecule cavity consisted of two whispering gallery mode (WGM) microcavities, where one WGM cavity is an optomechanical cavity with high-cavity dissipation *κ* and the other WGM cavity is an auxiliary ordinary optical cavity with high-quality factor (Q). Controlling the parameters of the system, such as the coupling strength *J* between the two cavities, the decay rate ratio *δ* of the two cavities, and the pump power *P*, the optical bistability can be controlled. Furthermore, the FWM process which presents the normal mode-splitting is also investigated in the FWM spectrum under different parameter regimes. Our study may provide a further insight of nonlinear phenomena in the composite photonic-molecule optomechanic systems.

## Background

Optomechanic systems (OMS) [1], consisting of optical cavities coupled to mechanical resonators and exploring radiation pressure-induced coherent photon-phonon interactions, have recently attracted much attention because they offer a platform to manipulate mechanical resonators and electromagnetic fields, and pave the way for potential applications of optomechanical devices, such as phonon laser [2, 3], sensing [4], phonon squeezing [5], the realization of squeezed light [6–8], ground-state cooling [9–11], and optomechanically induced transparency (OMIT) [12–15]-induced store light in solid-state devices [16, 17]. Although most attention has been paid to the single OMS, to realize compound OMS by integrating more optical or mechanical modes such as one mechanical mode coupled to two optical modes via radiation pressure [18, 19] and the phononic interaction between two mechanical resonators [20, 21] become a tendency for further investigating the OMS and their potential applications in quantum information processing. Based on the hybrid compound OMS, the transfer of a quantum state [22], OMIT-like phonon cooling [23], optomechanical dark mode [24], and phonon-mediated electromagnetically induced absorption [25] has been researched widely. In the numerous compound OMS, as a natural extension of the generic OMS, two directly coupled whispering gallery mode (WGM) microcavities termed photonic-molecule [26, 27] with optomechanical effect in one WGM microcavity have attracted much attention. There are two kinds of interplay in the compound photonic-molecule optomechanical system: the first one is the optomechanical interaction induced by the radiation pressure and the other one is cavity-cavity coupling via tunable photon tunneling. The two interactions together give rise to several interesting phenomena including phonon lasing [2, 3], chaos [28], ground-state cooling [23], and coherent control of light transmission [25, 29, 30].

On the other hand, OMS also provide a platform to investigate the nonlinear effect of light-matter interaction. Among all the nonlinear phenomena in OMS, optical bistability and four-wave mixing (FWM) are typical nonlinear optical phenomenon focusing on researchers’ interest. In recent year, the bistable behavior of the mean intracavity photon number has been extensively studied in various OMS, such as Bose-Einstein condensate cavity optomechanical system [31, 32], OMS with a quantum well [33], ultracold atoms [34, 35], and other hybrid OMS [36, 37]. In addition, FWM can be described as the cavity driven by a strong pump laser with frequency *ω*_*p*_ and a weak probe laser frequency *ω*_*s*_, and then, two pump photons would mix with a probe photon via the mechanical mode to yield an idler photon at frequency 2*ω*_*p*_−*ω*_*s*_ in OMS, and it is also investigated in previous works, such as the mode-splitting in strong coupling optomechanical system [38], coherent mechanical driving OMS [39, 40], and a two-mode cavity optomechanical system [41]. However, optical bistability and FWM have been seldom studied in composite photonic-molecule OMS, where the coupling strength represented by *J* of the two cavities play a key role affecting these nonlinear optical phenomena.

In the present work, we consider a composite photonic-molecule cavity optomechanical system, consisted of two WGM microcavities, where one WGM cavity is an optomechanical cavity with high-cavity dissipation *κ*, and the other WGM cavity is an auxiliary ordinary optical cavity with high-quality factor (Q) [42]. As Liu et al. [43] demonstrated, it remains difficult to achieve high Q factor and small mode volume (V) simultaneously for the same type of resonator. In the photonic-molecule optomechanics, by coupling the originally optomechanical cavity *c* with high-cavity dissipation *κ* (without high Q) to an auxiliary cavity mode *a* with high Q but large V, the requirement for high Q and small V for the same cavity can be removed. We introduce a ratio parameter *δ*=*κ*_*c*_/*κ*_*a*_, where *κ*_*c*_=*ω*_*c*_/*Q*_*c*_ and *κ*_*a*_=*ω*_*a*_/*Q*_*a*_ are the decay rates of cavity modes *c* and *a* (*ω*_*c*_ and *ω*_*a*_ are the frequencies of cavity *c* and *a*) to investigate the nonlinear effect in the photonic-molecule optomechanics. Here, the optomechanical cavity *c* is driven by the pump laser while the auxiliary cavity *a* is driven by the probe laser. The cavity *c* is coupled to cavity *a* via evanescent field, and the coupling strength *J* between the two cavities can be controlled by varying the separation between the two WGM cavities [26]. We investigate the optical bistability and FWM based on the composite photonic-molecule OMS by varying the coupling strength *J* between the cavity resonators, and a tunable and controllable optical bistability and FWM can be achieved with manipulating the coupling strength *J* between the two cavities. Further, with adjusting the parameter *δ* and the pump power *P*, the FWM process can be controlled.

## Model and Theory

**Fig. 1:**
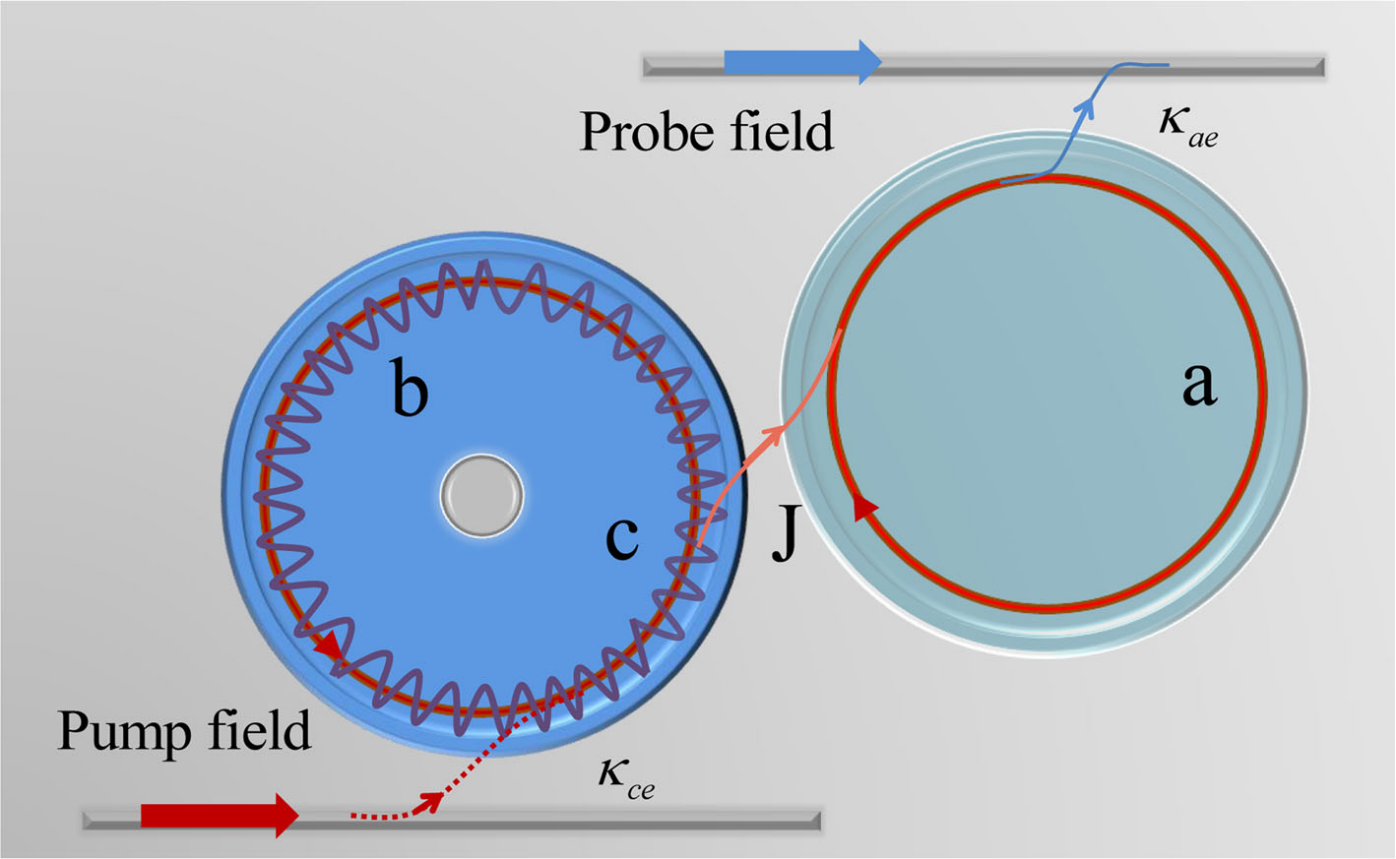
Schematic diagram of the composite photonic-molecule cavity optomechanical system including two WGM cavities. The first WGM cavity with high-cavity dissipation *κ* is optomechanical cavity *c* driven by a pump laser, and radiation pressure force induces the mechanical mode *b* coupling to cavity *c* with coupling strength *g*. The second WGM cavity *a* is an auxiliary cavity driven by a probe laser with high-quality factor (Q). The optomechanical cavity *c* is coupled to cavity *a* via evanescent field, and we introduce a parameter *J* to describe the coupling strength of the two cavities, which can be controlled by varying the separation between them [26]

The photonic-molecule optomechanics is shown in Fig. 1. The first cavity supports an optical mode *c* with the frequency *ω*_*c*_ driven by the pump laser with frequency *ω*_*p*_ and the amplitude $\varepsilon _{p}= \sqrt {P/\hbar \omega _{p}}$. The radiation pressure induces a mechanical mode *b* with the mechanical resonator frequency *ω*_*m*_, and the single-photon optomechanical coupling rate is *g*=*g*_0_*x*_0_ (*g*_0_=*ω*_*c*_/*R* and *R* is the radius of cavity *c*), and the zero-point fluctuation of the mechanical oscillator’s position is $x_{0}=\sqrt {\hbar /2M\omega _{m}} $ [13]. Then, the Hamiltonian of optomechanics *c* is [13]

1 $$ H_{c}=\hbar \Delta_{c}c^{\dag }c+\hbar \omega_{m}b^{\dag }b-\hbar ga^{\dag }a\left(b^{\dag }+b\right)+i\hbar \sqrt{\kappa_{ce}}\varepsilon_{p}\left(c^{\dag }-c\right),  $$

where *Δ*_*c*_=*ω*_*c*_−*ω*_*p*_ is the detuning of the pump field and cavity *c*. *c* and *c*^*†*^ represent the bosonic annihilation and creation operators of the cavity mode *c*, and *b*^*†*^(*b*) is the creation (annihilation) operator of mechanical mode. The auxiliary cavity only supports an optical mode *a* driven by the probe laser with frequency *ω*_*s*_, and its amplitude *ε*_*s*_ is $\varepsilon _{s}=\sqrt { P_{s}/\hbar \omega _{s}}$. We introduce the annihilation and creation operators *a* and *a*^*†*^ to describe the cavity *a*, and its Hamiltonian is [13]

2 $$ H_{a}=\hbar \Delta_{a}a^{\dag }a+i\hbar \sqrt{\kappa_{ae}}\varepsilon_{s}\left(a^{\dag }e^{-i\Omega t}-ae^{i\Omega t}\right)  $$

where *Δ*_*a*_=*ω*_*a*_−*ω*_*p*_ is the detuning of the pump field and cavity *a*, and *Ω*=*ω*_*s*_−*ω*_*p*_ is the pump-probe detuning. We use two tapered fibers to excite the cavity mode *a* and cavity mode *c* as the optical waveguide with the coupling rate *κ*_*ae*_ and *κ*_*ce*_. The optomechanical cavity *c* couples to cavity *a* through an evanescent field, and the cavity-cavity coupling rate *J* can be efficiently tuned by changing the distance between them [26]. When the coupling strength *J* is weak in between the two cavities, then the energy from cavity *c* cannot transfer easily to cavity *a*. Conversely, if the coupling strength *J* increases with decreasing the distance between the two cavities, then the energy can easily flow from the two cavities. The linearly coupled interaction between the two cavities is described by [26] $\hbar J\left (a^{\dag }c+ac^{\dag }\right)$. Then, the total Hamiltonian in the rotating wave frame of pump frequency *ω*_*c*_ can be written [3, 13, 23]

3 $$ \begin{aligned} H =&\hbar \Delta_{a}a^{\dag }a+\hbar \Delta_{c}c^{\dag }c+\hbar \omega_{m}b^{\dag }b+\hbar J\left(a^{\dag }c+ac^{\dag }\right)-\hbar ga^{\dag }a\left(b^{\dag }+b\right) \\ &+i\hbar \sqrt{\kappa_{ce}}\varepsilon_{p}\left(c^{\dag }-c\right)+i\hbar \sqrt{ \kappa_{ae}}\varepsilon_{s}\left(a^{\dag }e^{-i\Omega t}-ae^{i\Omega t}\right). \end{aligned}  $$

The decay rate of the two cavities mode *κ*=*κ*_*c*_=*κ*_*a*_=*κ*_*ex*_+*κ*_0_ with the intrinsic photon loss rate *κ*_0_, and *κ*_*ex*_ describes the rate at which energy leaves the optical cavity into propagating fields [13]. Here, for simplicity, we only consider the condition of *κ*_*ex*_=*κ*_0_=*κ*_*ae*_=*κ*_*ce*_, and we consider *ω*_*c*_=*ω*_*a*_.

We use the Heisenberg equation of motion $i\hbar \partial _{t}O=[O,H]$ (*O*=*a*,*c*,*X*) and introduce corresponding damping and noise operators, and we obtain the quantum Langevin equations as follows [44]:

4 $$ \partial_{t}a=-(i\Delta_{a}+\kappa_{a})a-iJc+\sqrt{\kappa_{ae}} \varepsilon_{s}e^{-i\Omega t}+\sqrt{2\kappa_{a}}a_{\text{in}},  $$

5 $$ \partial_{t}c=-(i\Delta_{c}+\kappa_{c})c+igcX-iJa+\sqrt{\kappa_{ce}} \varepsilon_{p}+\sqrt{2\kappa_{c}}c_{\text{in}},  $$

6 $$ \partial_{t}^{2}X+\gamma_{m}\partial_{t}X+\omega_{m}^{2}X=2g\omega_{m}c^{\dagger }c+\xi,  $$

where *X*=*b*^*†*^+*b* is the position operator and *γ*_*m*_ is the decay rate of the resonator. *a*_in_ and *c*_in_ describing the Langevin noises follow the relations [45]

7 $$\begin{array}{@{}rcl@{}} \left\langle a_{\text{in}}(t)a_{\text{in}}^{\dagger }\left(t^{^{\prime }}\right)\right\rangle &=&\left\langle c_{\text{in}}(t)c_{\text{in}}^{\dagger }\left(t^{^{\prime }}\right)\right\rangle =\delta\left(t-t^{^{\prime }}\right), \end{array} $$

8 $$\begin{array}{@{}rcl@{}} \left\langle a_{\text{in}}(t)\right\rangle &=&\left\langle c_{\text{in}}(t)\right\rangle =0. \end{array} $$

The resonator mode is influenced by stochastic force process with the following correlation function [46]

9 $$ \left\langle \xi^{\dagger }(t)\xi \left(t^{^{\prime }}\right)\right\rangle \,=\,\frac{ \gamma_{m}}{\omega_{m}}\int\! \frac{d\omega }{2\pi }\omega e^{-i\omega \left(t-t^{^{\prime }}\right)}\left[1\,+\,\coth \left(\frac{\hbar \omega }{2\kappa_{B}T}\right)\right],  $$

where *k*_*B*_ is Boltzmann constant and *T* indicates the reservoir temperature.

When the optomechanical cavity *c* is driven by a strong pump laser, the Heisenberg operator can be divided into two parts, i.e., steady-state mean value *O*_0_, and small fluctuation *δO* with zero mean value 〈*δO*〉=0. The steady-state values determine the intracavity photon numbers (*n*_*a*_=|*a*_*s*_|^2^and *n*_*c*_=|*c*_*s*_|^2^) determined by

10 $$ n_{c}=\frac{\kappa_{ce}\varepsilon_{p}^{2}\left(\Delta_{a}^{2}+\kappa_{a}^{2}\right) }{\left(\Delta^{^{\prime }2}+\kappa_{c}^{2}\right)\left(\Delta_{a}^{2}+\kappa_{a}^{2}\right)+2J^{2}\left(\kappa_{a}\kappa_{c}-\Delta^{^{\prime }}\Delta_{a}\right)+J^{4}},  $$

11 $$ n_{a}=\frac{\kappa_{ce}\varepsilon_{p}^{2}J^{2}}{\left(\Delta^{^{\prime }2}+\kappa_{c}^{2}\right)\left(\Delta_{a}^{2}+\kappa_{a}^{2}\right)+2J^{2}\left(\kappa_{a}\kappa_{c}-\Delta^{^{\prime }}\Delta_{a}\right)+J^{4}},  $$

where $\Delta ^{^{\prime }}=\Delta _{c}-2g^{2}n_{c}/\omega _{m}$. This form of coupled equations are characteristic of the optical bistability. In the following section, we will discuss the parameters such as the pump power *P*, the cavity-cavity coupling strength *J*, and the ratio parameter *δ* that affect the optical bistability. Keeping only the linear terms of the fluctuation operators and making the ansatz [47] 〈*δa*〉=*a*_+_*e*^−*iΩt*^+*a*_−_*e*^*iΩt*^, 〈*δc*〉=*c*_+_*e*^−*iΩt*^+*c*_−_*e*^*iΩt*^, 〈*δX*〉=*X*_+_*e*^−*iΩt*^+*X*_−_*e*^*iΩt*^, we then obtain

12 $$ a_{-}=\frac{\Lambda_{1}}{\Lambda_{2}-\Lambda_{3}},  $$

where $\Lambda _{1}=igc_{s}^{2}\eta ^{\ast }J^{2}\varepsilon _{s}\sqrt { \kappa _{ae}}$, *Λ*_2_=(*iΔ*_*a*2_+*κ*_*a*_)(*iΔ*_2_+*κ*_*c*_)[(*iΔ*_1_−*κ*_*c*_)(*iΔ*_*a*1_−*κ*_*a*_)−*J*^2^], $\Lambda _{3}=-g^{2}\eta ^{\ast 2}n_{c}^{2}(i\Delta _{a1}-\kappa _{a})(i\Delta _{a2}+\kappa _{a})$, *Δ*_*a*1_=*Δ*_*a*_−*Ω*, *Δ*_*a*2_=*Δ*_*a*_+*Ω*, $\Delta _{1}=\Delta ^{^{\prime }}-\Omega +g\eta n_{c}$, $\Delta _{2}=\Delta ^{^{\prime }}+\Omega +g\eta ^{\ast }n_{c}$, and $\eta =2g\omega _{m}/(\omega _{m}^{2}-i\gamma _{m}\Omega -\Omega ^{2})$. Using the standard input-output relation [45] $a_{\text {out}}(t)=a_{\text {in}}(t)-\sqrt {2\kappa _{a}}a(t)$, where *a*_out_(*t*) is the output field operator, and obtain the expectation value of the output fields:

13 $$ {\begin{aligned}  a_{\text{out}}(t)&=(\varepsilon_{p}-\sqrt{\kappa_{ae}}a_{s})e^{-i\omega_{p}t}+(\varepsilon_{s}-\sqrt{\kappa_{ae}}a_{+})e^{-i(\delta +\omega_{p})t}-\sqrt{\kappa_{ae}}a_{-}e^{-i(\delta -\omega_{p})t} \\ &=(\varepsilon_{p}-\sqrt{\kappa_{ae}}a_{s})e^{-i\omega_{p}t}+(\varepsilon_{s}-\sqrt{\kappa_{ae}}a_{+})e^{-i\omega_{s}t}-\sqrt{\kappa_{ae}} a_{-}e^{-i(2\omega_{p}-\omega_{s})t} \end{aligned}}  $$

where *a*_out_(*t*) is the output field operator. Equation (13) shows that the output field consists of three terms. The first term corresponds to the output field at driving field with amplitude *ε*_*p*_ and frequency *ω*_*p*_. The second term corresponds to the probe field with frequency *ω*_*s*_ related to the anti-Stokes field resulting in OMIT, which has been investigated in various optomechanical systems [12–15, 48]. The last one corresponds to the output field with frequency 2 *ω*_*p*_−*ω*_*s*_ related to the stoke field displaying the FWM. In the FWM process, the two photons of the driving field interact with a single photon of the probe field each with frequencies *ω*_*p*_ and *ω*_*s*_ born a new photon of frequency 2 *ω*_*p*_−*ω*_*s*_. The FWM intensity in terms of the probe field can be defined as [49]

14 $$ \text{FWM}=\left\vert \frac{\sqrt{\kappa_{ae}}a_{-}}{\varepsilon_{s}}\right\vert^{2}\text{,}  $$

which is determined by the optomechanical coupling strength *g*, the pump power *P*, the cavity-cavity coupling strength *J*, and the decay rate ratio *δ* of the two cavities.

## Numerical Results and Discussions

In this section, we first investigate the bistable behavior of the steady-state photon number *n*_*c*_ and *n*_*a*_ of the two cavities according to Eqs. (10) and (11). Because it is too cumbersome to give the analytical expression of the bistability condition, here we will present the numerical results. We choose the parameters similar to those in Ref. [13, 26] : the parameters of cavity *c* as [13]: *g*_0_=12 GHz/nm, *γ*_*m*_=41 kHz, *ω*_*m*_=51.8 MHz, *κ*_*c*_=5 MHz, *m*=20 ng, *λ*=750 nm, and *Q*=1500, and the order of magnitude of the pump power is milliwatt (1 mW =10^−3^ W). For cavity *a*, we consider *ω*_*a*_=*ω*_*c*_ and *κ*_*c*_=*κ*_*a*_. The coupling strength *J* between the two cavity modes plays a key role and can affect the bistable behavior and FWM. It has been reported experimentally that the coupling strength *J* depends on the distance between cavity *c* and cavity *a* [26] (also the coupling strength decreases exponentially with increasing the distance of the two cavities). Here, we expect the coupling strength $ J\sim \sqrt {\kappa _{c}\kappa _{a}}$.

**Fig. 2:**
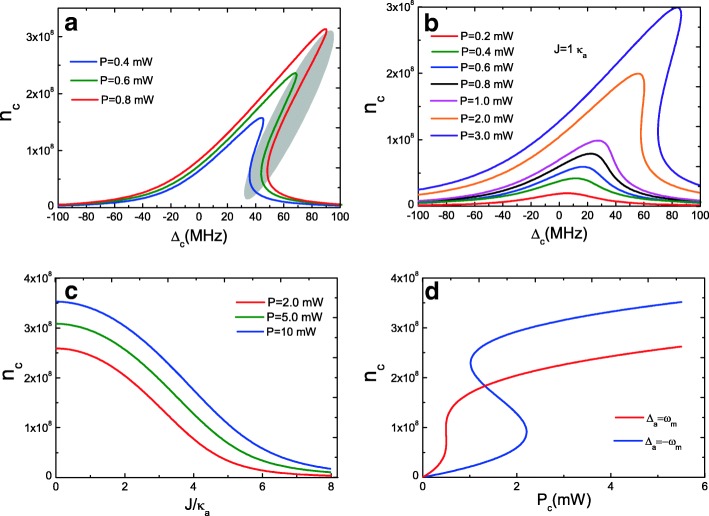
**a** Mean intracavity photon number of optomechanical cavity *c* as a function of the cavity-pump detuning *Δ*_*c*_ with three pump powers at *J*=0. **b** Mean intracavity photon number of optomechanical cavity *c* as a function of the cavity-pump detuning *Δ*_*c*_ with several different pump powers under *J*=1.0 *κ*_*a*_. **c** Mean intracavity photon number *n*_*c*_ of optomechanical cavity *c* as a function of *J* with three pump powers. **d** Mean intracavity photon number *n*_*c*_ as a function of *P* for *Δ*_*c*_=*Δ*_*a*_=*ω*_*m*_

Equations (10) and (11) giving the intracavity photon numbers of optomechanical cavity *c* and ordinary cavity *a* are coupled cubic equations, which exhibit bistable behavior. We first consider the condition of *J*=0, i.e., only a single optomechanical cavity *c*, and Fig. 2a plots the mean intracavity photon number *n*_*c*_ of optomechanical cavity *c* as a function of the cavity-pump detuning *Δ*_*c*_=*ω*_*c*_−*ω*_*p*_ with three pump powers. When the pump power is less than *P*=0.4 mW (such as *P*=0.1 mW), the curve is nearly Lorentzian. With increasing the power *P* to a critical value, the optomechanical cavity *c* exhibits bistable behavior, as shown in the curves for *P*=0.4 mW to *P*=0.8 mW, where the initially Lorentzian resonance curve becomes asymmetric. The mean intracavity photon number *n*_*c*_ has three real roots (Eq. (10)), and the largest and smallest roots are stable, and the middle one is unstable, which is represented in an oval in Fig. 2a. However, when we consider the optical cavity *a*, i.e., *J*≠0 such as *J*=1.0 *κ*_*a*_, the bistable behavior is broken in some ways as shown in Fig. 2b. That is because when optomechanical cavity *c* coupled to optical cavity *a*, parts of intracavity photon number *n*_*c*_ of optomechanical cavity *c* will coupled into optical cavity *a*, and therefore, intracavity photon number *n*_*c*_ will decrease and then result in a destroyed bistable behavior. Figure 2c shows the mean intracavity photon number *n*_*c*_ of optomechanical cavity *c* as a function of the cavity-cavity coupling strength *J* with three pump powers. Obviously, the mean intracavity photon number *n*_*c*_ depends on the pump power *P*, and the intracavity photon number *n*_*c*_ is always decreasing with the increasing coupling strength *J* because parts of photon number are coupled into optical cavity *a*. Further, larger cavity-pump detuning is beneficial to observe the optical bistable behavior with increasing pump power *P*. Figure 2d plots the mean intracavity photon number *n*_*c*_ versus the pump power *P* with cavity *a* at red sidebands (*Δ*_*a*_=*ω*_*m*_) and blue sidebands (*Δ*_*a*_=−*ω*_*m*_), respectively, and the bistability presents the hysteresis loop behavior [50]. However, our results are different from the previous work of two-mode optomechanical system without considering the cavity-cavity coupling *J*. Therefore, the coupling strength *J* plays an important role in the bistability.

**Fig. 3:**
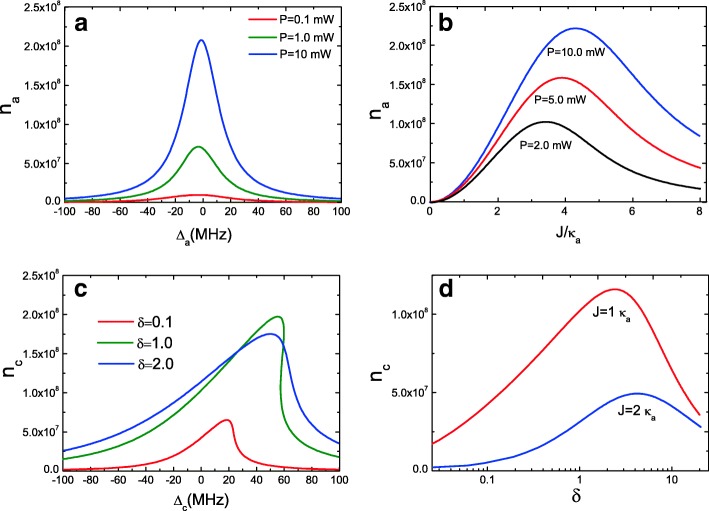
**a** Mean intracavity photon number of ordinary cavity *a* as a function of the cavity-pump detuning *Δ*_*a*_ with three pump powers at *J*=1.0 *κ*_*a*_. **b** Mean intracavity photon number *n*_*a*_ as a function of *J* with three pump powers. **c** Mean intracavity photon number *n*_*c*_ as a function of *Δ*_*c*_ with three ratio parameters *δ*. **d** Mean intracavity photon number *n*_*c*_ as a function of *δ* for two *J*

We further investigate bistable behavior of optical cavity *a* with Eq. (11). Figure 3a gives the intracavity photon number *n*_*a*_ of ordinary cavity *a* as a function of the cavity-pump detuning *Δ*_*a*_=*ω*_*a*_−*ω*_*p*_ with pump powers *P*=0.1 mW, *P*=1.0 mW, and *P*=10 mW at *J*=1.0 *κ*_*a*_. It is obvious that optical cavity *a* cannot behave as bistable behavior due to intracavity photon number *n*_*a*_ of cavity *a* from cavity *c* cannot maintain bistability in low-pump power. Actually, only high-pump power *P* can cavity *a* present bistable behavior, because only high-pump power driven optomechanical cavity *c*, much more photon number can couple into optical cavity *a*. We also plot the mean intracavity photon number *n*_*a*_ of optical cavity *a* as a function of the coupling strength *J* under three pump powers as shown in Fig. 3b. It is clear that when *J*=0, *n*_*a*_=0, because there is no coupling between the two cavities at *J*=0, and at this condition, no photon couples into optical cavity *a*. With increasing the coupling strength *J* (decreasing the distance of the two cavities [26]), the intracavity photon numbers *n*_*a*_ of ordinary optical cavity *a* increase but not always. There is an optimum coupling strength *J* for the maximum value of *n*_*a*_ under different pump power, and then, *n*_*a*_ will decrease with the increasing *J*. It is a remarkable fact that the coupling strength *J* between the two cavities can be adjusted [26].

In addition, we consider a ratio parameter *δ*=*κ*_*c*_/*κ*_*a*_ (*κ*_*c*_=*ω*_*c*_/*Q*_*c*_ and *κ*_*a*_=*ω*_*a*_/*Q*_*a*_) to investigate the parameters of the two cavities that influence bistable behavior. *κ* is the decay rate of the cavity mode, which is related to the frequency and quality factor of the cavity. As we know, it is difficult to achieve high Q and small V simultaneously for a cavity mode due to the diffraction limit. For an optical cavity, a smaller V corresponding to a larger radiative decay rate results in a lower Q. Although different types of cavities possess their own unique properties, the weigh between high Q and small V still exists. However, when by coupling the originally OMS *c* with high-cavity dissipation to an auxiliary cavity mode *a* with high Q but large V, the bistable behavior will change significantly. Figure 3c shows the mean intracavity photon number *n*_*c*_ of optomechanical cavity *c* as a function of *Δ*_*a*_ under several different *δ*=*κ*_*c*_/*κ*_*a*_ with an unchanged coupling strength *J*=1.0 *κ*_*a*_. We can find that the bistable behavior can appear, but the intracavity photon number *n*_*c*_ is small at *δ*=0.1 with *J*=2 *κ*_*a*_, i.e., *κ*_*c*_=0.1 *κ*_*a*_ which means *Q*_*c*_>*Q*_*a*_. When increasing the ratio *δ* from *δ*=1.0 to *δ*=2.0, the intracavity photon number *n*_*c*_ experiences the change from bistable behavior to nearly Lorentzian line profile. That is to say when *Q*_*c*_<*Q*_*a*_, the bistable behavior will be broken, but there is an optimal condition, i.e., *Q*_*c*_=*Q*_*a*_. In Fig. 3d, we give the intracavity photon number *n*_*c*_ as a function of *δ* with two different *J*, and obviously, in increasing the ratio parameter *δ*, the intracavity photon numbers *n*_*c*_ increase. When it reaches an optimum value for a given *J*, then *n*_*c*_ decrease. Therefore, controlling the cavity parameters, like the decay rate *κ* or the quality factor of the cavities, the bistable behavior can be controlled.

**Fig. 4:**
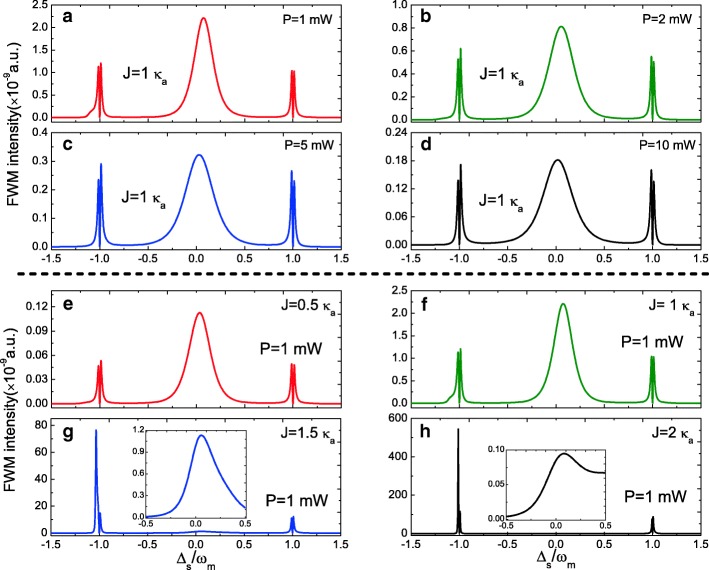
**a**–**d** FWM intensity as a function of the normalized probe-pump detuning *Δ*_*s*_ for different pump power at *J*=1.0 *κ*_*a*_. **e**–**h** FWM intensity as a function of *Δ*_*s*_ for different *J* at pump power *P*=1.0 mW

On the other hand, as a typical nonlinear optical phenomenon, we also investigate the FWM process with Eq. (14) in the photonic-molecule optomechanical system. Figure 4 plots the FWM spectrum as a function of the probe-cavity *a* detuning *Δ*_*s*_=*ω*_*s*_−*ω*_*a*_ at *Δ*_*a*_=*Δ*_*c*_=0 under different parameter regimes. Figure 4a–d displays the FWM spectra evolution under different pump power *P* at *J*=1.0 *κ*_*a*_. It is clear that the FWM spectra present three peaks, where a Lorentzian peak near *Δ*_*s*_=0 and two mode-splitting peaks locate at ±*ω*_*m*_, and the FWM intensity decreases with increasing the pump power. Figure 4e–h shows the change of FWM spectra from *J*=0.5 *κ*_*a*_ to *J*=2.0 *κ*_*a*_ at pump power *P*=1.0 mW. With increasing the coupling strength *J* from *J*=0.5 *κ*_*a*_ to *J*=2.0 *κ*_*a*_, the FWM spectra change significantly. The phenomena can be explained with a dressed-state picture which has demonstrated in single cavity optomechanical system [51].

**Fig. 5:**
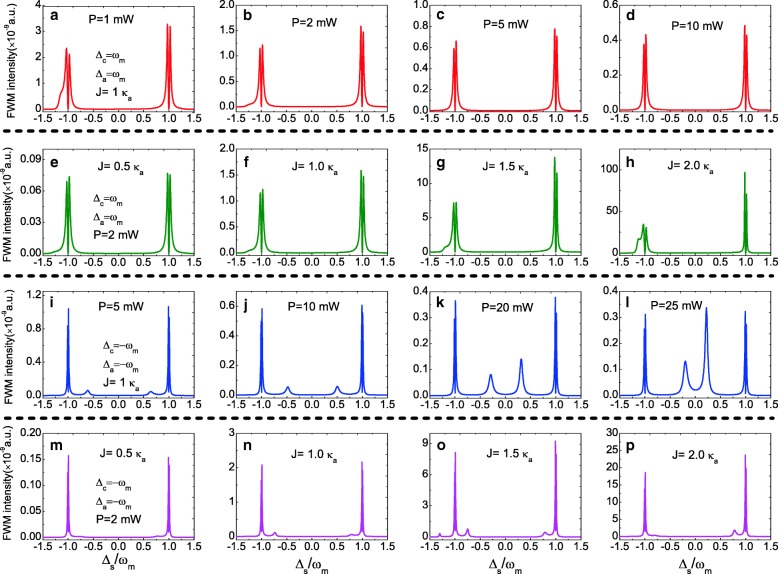
**a**–**d** FWM intensity as a function of *Δ*_*s*_ for different pump power *P* at the red sideband (*Δ*_*c*_=*Δ*_*a*_=*ω*_*m*_) and *J*=1.0 *κ*_*a*_. **e**–**h** FWM intensity as a function of *Δ*_*s*_ for different *J* under the red sideband and the pump power *P*=2.0 mW. **i**–**l** FWM intensity as a function of *Δ*_*s*_ for different pump power *P* at the blue sideband (*Δ*_*c*_=*Δ*_*a*_=−*ω*_*m*_) and *J*=1.0 *κ*_*a*_. **m**–**p** FWM intensity as a function of *Δ*_*s*_ for different *J* under the blue sideband and the pump power *P*=2.0 mW

We then investigate the FWM spectra at *Δ*_*a*_=*Δ*_*c*_≠0. Figure 5a–d gives the FWM spectra at the red sideband, i.e., *Δ*_*a*_=*Δ*_*c*_=*ω*_*m*_ under an unchanged *J*=1.0 *κ*_*a*_ with increasing the pump power from *P*=1.0 to *P*=10 mW. Two normal mode-splitting peaks appear in the FWM spectra locating at ±*ω*_*m*_ respectively, and the FWM intensity decreases with increasing the pump power. Figure 5e–h shows the FWM spectra at the red sideband, i.e., *Δ*_*a*_=*Δ*_*c*_=*ω*_*m*_ under a fixed pump power *P*=2.0 mW with increasing the coupling strength *J* from *J*=0.5 *κ*_*a*_ to *J*=2.0 *κ*_*a*_. Obviously, the FWM intensity increases with increasing the coupling strength *J*, and the bigger *J* means more photon numbers coupled into optical cavity *a*. When changing the detuning *Δ*_*a*_ and *Δ*_*c*_ from the red sideband to the blue sideband, i.e., *Δ*_*a*_=*Δ*_*c*_=−*ω*_*m*_, the evolution of the FWM spectra change prominently. Figure 5i–l displays the FWM spectra at blue sideband under four different pump powers, and the FWM intensity decreases with increasing the pump power even at the blue sideband. Except two normal mode-splitting peaks locating at ±*ω*_*m*_, there are also two sharp sideband peaks appear in the FWM spectra and their location are related to the pump power. In Fig. 5m–p, we also discuss the coupling strength *J* that affect the FWM spectra under the blue sideband. Whether other sharp sideband peaks appear in the FWM spectra depend on the the coupling strength *J*.

**Fig. 6:**
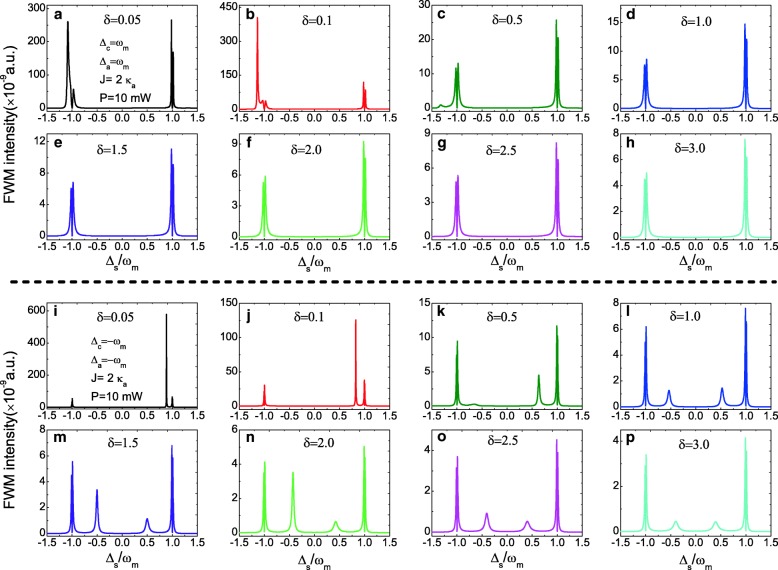
**a**–**h** FWM intensity as a function of *Δ*_*s*_ for several different ratio parameters *δ* at the red sideband (*Δ*_*c*_=*Δ*_*a*_=*ω*_*m*_) and *J*=2.0 *κ*_*a*_, *P*=10 mW. **i**–**p** FWM intensity as a function of *Δ*_*s*_ for several different ratio parameters *δ* at the blue sideband (*Δ*_*c*_=*Δ*_*a*_=−*ω*_*m*_) and *J*=2.0 *κ*_*a*_, *P*=10 mW

Further, since the ratio parameter *δ*=*κ*_*c*_/*κ*_*a*_ can influence the intracavity photon number in the composite photonic-molecule OMS, the FWM spectra can be manipulated with controlling the parameter *δ*. Figure 6a–h presents the FWM spectra at unchanged parameters *J*=2.0 *κ*_*a*_ and *P*=10 mW under the red sideband with increasing the ratio *δ* from *δ*=0.05 to *δ*=3.0, and the FWM intensity decreases with increasing the ratio *δ*. While in the blue sideband, other sharp sideband peaks will appear in the FWM spectra as shown in Fig. 6i–p, and the FWM intensity also decreases with increasing the ratio *δ*. Therefore, with controlling the cavity parameters, like the decay rate *κ* or the Q of the cavities, the FWM can achieve straightforward in the composite photonic-molecule OMS.

## Conclusion

We have investigated the optical bistability and four-wave mixing in a composite WGM cavity photonic-molecule optomechanical system, which includes an optomechanical cavity with high-cavity dissipation coupled to an auxiliary cavity with high-quality factor. We investigate the optical bistability under different parameter regimes such as the coupling strength *J* between the two cavities and the decay rate ratio *δ* of the two cavities in the system. The optical bistability can be adjusted by the pump field driving the optomechanical cavity, and the intracavity photon number in the two cavities is determined by the coupling strength *J*. Further, we have also demonstrated how to control the FWM process in the photonic-molecule optomechanical system under different driving conditions (the red sideband and the blue sideband) and different parameter conditions (the coupling strength *J* and the ratio *δ*). Numerical results show that the FWM process can be controlled with such parameters. These results are beneficial for better understanding the nonlinear phenomena in the composite photonic-molecule optomechanical system.

## Abbreviations

C-OMS: Cavity optomechanics systems
FWM: Four-wave mixing
OMS: Optomechanics systems
OMIT: Optomechanically induced transparency
Q: Quality
V: Volume
WGM: Whispering gallery mode
